# Multiple Phantom Tumor of the Lung: A Complex Appearance Resolving with Appropriate Intervention

**Published:** 2016

**Authors:** Haleh Mikaeili, Jafar Mehdizadeh Baghbani

**Affiliations:** Tuberculosis and Lung Diseases Research Centre, Tabriz University of Medical Sciences, Tabriz, Iran.

**Keywords:** Pleural effusion, Congestive heart failure, Diagnosis

## Abstract

The term phantom tumor may be used to describe a well-demarcated opacity resulted from pleural effusion. Phantom tumors are commonly associated with congestive heart failure causing transudative pleural effusion within pulmonary fissures. The figure may bring about inaccurate invasive diagnostic interventions. We report a heavy smoker patient with multiple phantom tumors in the right lung resolved with medical management. This case report provides records for a timely management of similar patients.

## INTRODUCTION

Fluid collection in minor and major fissures of the lungs resulted from congestive heart failure or chronic renal failure, is not a common finding on chest roentgenograms. But proper diagnosis is essential to commence the appropriate treatment and avoid inaccurate interventions.

This finding which is known as “phantom tumor” or “vanishing tumor” may be mistaken for lung mass or consolidation. Although it resolves with timely diagnosis and optimal treatment, previous experiments show that misdiagnosis may result in unnecessary lung biopsy or long-term antibiotic therapy (1,2). Reporting such cases emphasizes that it should always be considered as a differential diagnosis to facilitate diagnosis and management of these patients (3).

## CASE SUMMARIES

Our patient was an 80-year-old male with an established diagnosis of congestive heart failure, admitted to Shahid Madani Hospital affiliated to Tabriz University of Medical Sciences, with the chief complaint of exacerbation of dyspnea in the recent 10 days.

A comprehensive history was taken which was compatible with the New York Heart Association (NYHA) Functional Classification of IV. He was a heavy smoker smoking approximately 50 packs/year and presented with dry cough and shortness of breath. An echocardiography revealed ejection fraction of 20–25%. The patient underwent treatment for decompensated heart failure and an external pacemaker was installed because of hemodynamically unstable bradycardia.

**Figure 1.**
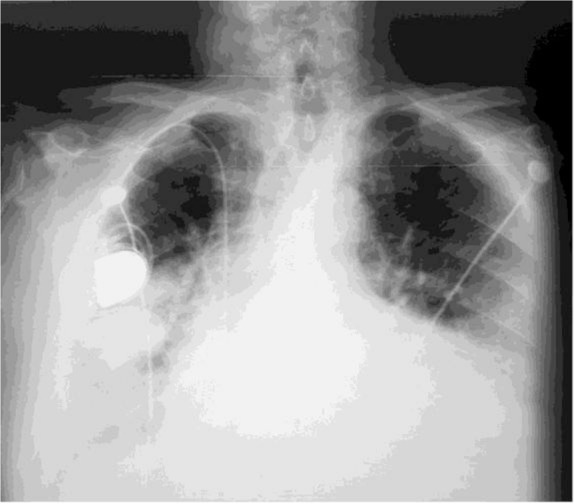
Chest X-ray showing three mass-like opacities in the right lung

Chest X-ray showed three mass-like opacities in the right lung (Figure 1). A pulmonary consultation was requested because of the abnormal findings on chest images and the significant history of heavy smoking. This recognized risk factor ranked neoplasms at the top of differential diagnosis list.

**Figure 2.**
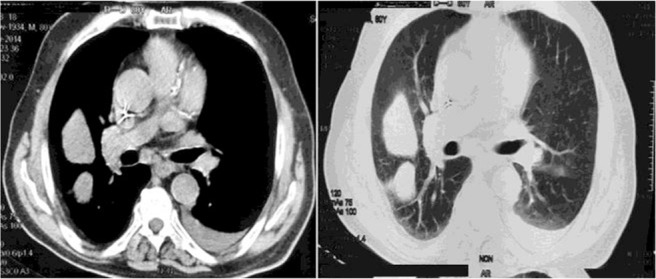
Computed tomography images showing bilateral plural effusion and three mass-like densities in the right lung

During the visit by a pulmonologist, the patient was awake and oriented and vital signs were stable. Dyspnea improved after several days of diuretic therapy. Pulmonary sounds decreased in the base of both lungs. Subsequently, spiral chest computed tomography was performed for further evaluations (Figure 2). Bilateral plural effusion and three mass-like densities in the right lung were reported. One of the masses seemed to be fluid collection in the right major fissure and the other two seemed to be fluid collection in the superior part of the major and minor fissures.

**Figure 3.**
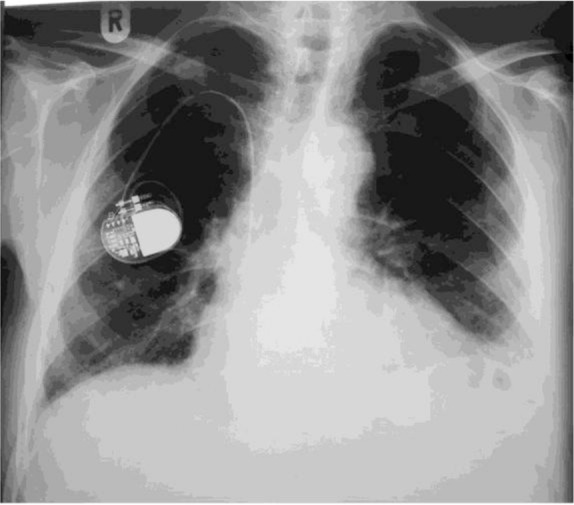
Repeat chest X-ray after 4 days of treatment for heart failure, showing clearance of lung field

Optimal treatment for heart failure and full dose diuretic therapy were recommended as well as a repeated chest X-ray in the following days. Four days later, a chest X-ray was obtained revealing clearance of the lung field, confirming the diagnosis of phantom tumor (Figure 3).

## DISCUSSION

Phantom or vanishing tumor is a rare but well-known condition (4, 5). Recognition of this radiographic finding is important because with an appropriate diagnosis, it vanishes following mobilization of the fluid and effective diuresis.

As described here too, these opacities may be more than one (5) and are mostly seen in the geriatric population (2) who have higher incidence of congestive heart failure. According to previous reports, it is not restricted to severe failure (6) and should be considered in all patients presenting with congestive heart failure with the described chest X ray pattern.

The most commonly described appearance is a well defined homogeneous mass, occupying the transverse fissure or sometimes the oblique interlobar fissures. As also described in the present case, fluid loculation has been in the right side in the majority of previous reports (4). In patients with congestive heart failure, greater hydrostatic pressure in the right side of the lung interferes with venous and lymphatic drainage and results in right side dominance of effusion (7). Additionally, pleuritis results in obliteration of the space and prevents spread of fluid (8). Phantom tumors are commonly found within the minor fissure, but as in the present case, they can occur within the major fissure as well (9).

Pulmonary infarction, pneumonia, tuberculosis, lobe collapse, malignant mass or metastatic neoplasm, abscess, emphysema, cyst and arteriovenous aneurysm are the differential diagnosis for this radiographic finding. Such case reports can make the diagnosis easier for internists and prevent invasive, unnecessary and costly interventions.

In conclusion, pleural effusion within pulmonary fissures may be presented as phantom tumor and a timely management will prevent unnecessary interventions.
